# Trajectory of migraine-related disability following long-term treatment with lasmiditan: results of the GLADIATOR study

**DOI:** 10.1186/s10194-020-01088-4

**Published:** 2020-02-24

**Authors:** Richard B. Lipton, Louise Lombard, Dustin D. Ruff, John H. Krege, Li Shen Loo, Andrew Buchanan, Thomas E. Melby, Dawn C. Buse

**Affiliations:** 1 Montefiore Medical Center, Bronx, NY USA; 2 Department of Neurology, Albert Einstein College of Medicine, Bronx, NY USA; 3 Eli Lilly and Company, Indianapolis, IN USA; 4 Syneos Health, Inc, Morrisville, NC USA

**Keywords:** Migraine, Serotonin, 5-HT_1F_ agonist, Ditan, MIDAS, Disability, Function, Presenteeism, Absenteeism

## Abstract

**Background:**

Migraine is recognized as the second leading cause of disability globally. Lasmiditan is a novel, selective serotonin 5-HT_1F_ receptor agonist developed for acute treatment of migraine. Here we analyzed effects of lasmiditan on migraine disability assessed with the Migraine Disability Assessment (MIDAS) scale for interim data from a long-term safety study.

**Methods:**

Completers of two single-attack parent studies were offered participation in the 1 year GLADIATOR study, that randomized participants to treatment with lasmiditan 100 mg or 200 mg taken as needed for migraine attacks of at least moderate severity. Changes in MIDAS were modeled using a mixed model repeated measures analysis.

**Results:**

The sample included 1978 patients who received ≥1 lasmiditan dose and were followed for a median of 288 days. Baseline mean MIDAS scores for the lasmiditan 100-mg and 200-mg groups were 29.4 and 28.9, respectively, indicating severe migraine-related disability. Relative to baseline, MIDAS total scores were significantly lower at 3, 6, 9, and 12 months for both dose groups. At 12 months, changes in MIDAS scores were − 12.5 and − 12.2 for lasmiditan 100 mg and 200 mg, respectively, with 49% and 53% of patients, respectively, achieving at least a 50% decrease in MIDAS total score. Statistically significant improvements were also seen for work and/or school absenteeism and presenteeism, monthly headache days, and mean headache pain intensity at all time points up to 1 year. Findings for patients who completed all visits versus those dropping out early were similar. Responses were generally similar for the lasmiditan 100 mg or 200 mg doses, between subgroups defined based on the number of baseline monthly migraine attacks (≤5 vs. >5), and also between subgroups defined by pain-free response (yes/no) during initial attacks.

**Conclusions:**

Long-term treatment with lasmiditan was associated with significant reductions in migraine-related disability, including both work or school absenteeism and presenteeism. The similarity of responses in completers and those who dropped out suggests that selective attrition does not account for the improvements. Benefits were significant at 3 months and maintained through 12 months.

**Trial registration:**

clinicaltrials.govNCT02565186; first posted October 1, 2015.

## Background

Migraine is a common and disabling disorder, affecting about 12% of the US population overall and 17% of women, with the highest prevalence between the ages of 18 and 55 [1–3]. Migraine is also highly prevalent and is recognized as the second leading cause of disability globally [4], and as the foremost cause of years lived with disability in people aged 15 to 49 [5].

Lasmiditan is an oral, selective 5-HT_1F_ receptor agonist indicated for the acute treatment of migraine. Lasmiditan is centrally penetrant and evidence suggests that lasmiditan exerts its therapeutic effects in the treatment of migraine by decreasing neuropeptide release and inhibiting pain pathways, potentially including both the trigeminal nerve innervation of the meningeal artery and in the trigeminocervical complex [6]. Lasmiditan lacks the vasoconstrictor activity which arises from the 5-HT_1B_ effects of triptans [6–10].

Two completed placebo-controlled Phase 3 studies assessed lasmiditan for the acute treatment of migraine: SAMURAI (*N* = 1856, ClinicalTrials.gov identifier: NCT02439320) and SPARTAN (*N* = 2583; NCT02605174) [11, 12]. The studies were similarly designed, double-blind, and placebo-controlled, with participants randomized to treat a single migraine attack with oral lasmiditan (50 mg [SPARTAN only], 100 mg, or 200 mg) or placebo. In both of those studies, all doses of lasmiditan were significantly more effective than placebo based on the primary endpoint, pain freedom at 2 h postdose, and the key secondary endpoint, freedom from the patient-designated most bothersome symptom at 2 h postdose. Lasmiditan was generally safe and well tolerated, with no deaths and few serious adverse events reported [13].

The GLADIATOR study is a prospective, randomized, open-label, Phase 3 study designed to assess the long-term safety of lasmiditan with inclusion of efficacy measures, including migraine disability. Participants were randomized to lasmiditan 100 mg and 200 mg for the treatment of multiple migraine attacks for up to 1 year. Patients in the current analysis had previously completed one of two Phase 3 single-attack pivotal efficacy studies [11, 12].

Data assessing the interim efficacy and safety and tolerability of lasmiditan in GLADIATOR have been reported previously, including overall data for MIDAS total score and headache days [14]. Specifically, at baseline, mean (Migraine Disability Assessment) MIDAS score was approximately 29, indicating, on average, severe migraine disability. Mean MIDAS total scores and number of days with headache decreased significantly from baseline at all timepoints for both lasmiditan treatment arms. In the present analyses of the same interim dataset, we further assessed changes in MIDAS total score, mean headache days, and average headache pain intensity across baseline patient characteristics and response subgroups, and assessed changes in absenteeism and presenteeism in the overall population.

## Methods

### Study design and participants

Patients who completed the pivotal efficacy studies (SAMURAI or SPARTAN) were potentially eligible to participate in the long-term safety study (GLADIATOR). Patients had episodic migraine, defined as 3–8 attacks/month and <15 monthly headache days at baseline, and fulfilled ICHD-2 criteria 1.1 or 1.2.1 for migraine with or without aura [15]. These studies included patients with cardiovascular risk factors according to American College of Cardiology/American Heart Association Task Force on Practice Guidelines [16], including age, total and high-density lipoprotein cholesterol, systolic blood pressure, diabetes, and current smoking status. SPARTAN did not exclude patients with uncontrolled hypertension, clinically significant arrhythmia, or known coronary artery disease, although these patients were excluded from SAMURAI.

The study design of GLADIATOR has been reported in detail previously [14] and is described in brief below. All participants provided written informed consent and confirmed that they were willing to complete an e-diary. The study was conducted in compliance with the International Council for Harmonization principles of Good Clinical Practice. Ethics review boards approved the study protocol and the informed consent form prior to study commencement. GLADIATOR was conducted at study sites in the United States, United Kingdom, and Germany.

#### Study design

Patients in GLADIATOR were randomized 1:1 to treatment with lasmiditan 100 mg or 200 mg, stratified (yes or no) for use of concomitant migraine preventive medications. Because of an insufficient supply of the 100-mg dose in Europe, all patients at European study sites were assigned to lasmiditan 200 mg.

Patients were instructed to use lasmiditan as the first treatment for each new migraine attack within 4 h of pain onset, provided that pain was moderate or severe and not improving. If migraine pain did not improve within 2 h, or if the pain resolved but recurred, a second dose of lasmiditan was allowed within 24 h of the first dose, as long as no other migraine medication had been used. Alternately, patients could take their own medication for rescue or recurrence within 2 to 24 h from first dose, although triptans, ergots, opioids, and barbiturates were not permitted within 24 h of lasmiditan administration. Patients were originally dispensed lasmiditan at up to 8 doses per month (24 doses for 3 month periods) in addition to doses remaining from the previous study period, with additional doses available upon request.

#### Data collection and endpoints

At the GLADIATOR screening visit, patients were trained on use of the e-diary and completed the MIDAS (see Additional file) [17, 18]. MIDAS is scored based on a 5-item questionnaire that measures lost time over the previous 3 months due to migraine in the domains of school and work, household work, and family, social, or non-work activities [17, 18]. Two additional questions are included regarding the average number of headache days and average headache pain over the last 3 months; however, these do not contribute to the MIDAS total score. MIDAS has demonstrated reliability and validity [17, 18] and scores correlate with clinical judgement on the need for medical care [19].

Patients were instructed to use an e-diary to record the details of each migraine attack. Patients were asked to record migraine headache pain using the 4-point pain severity rating scale (none, mild pain, moderate pain, and severe pain) at 0 (predose), 0.5, 1, 2, 4, 24, and 48 h postdose [20]. Pain freedom was defined as a reduction in pain severity from mild, moderate, or severe at baseline to none at the summarized time point. Migraine-related disability over the course of the attack was assessed with the e-diary question “How much is your migraine interfering with your normal activities” at 0 (predose), 0.5, 1, 2, 4, 24, and 48 h postdose. The 4 response options were “not at all” (0), “mild interference” (1), “marked interference” (2), and “need complete bed rest” (3). Baseline monthly migraine attacks were per study personnel entry on the case report form.

Other assessments included change from baseline in MIDAS total score, number of days with headache over the past 3 months, and average headache pain intensity over the last 3 months at 1, 3, 6, 9, and 12 months. Absenteeism days (missed days at work or school, see Additional file) were assessed based on MIDAS question 1 and presenteeism days (days present at work or school with substantially impaired productivity) were assessed based on MIDAS question 2 at 1, 3, 6, 9, and 12 months.

Clinic visits occurred at 1, 3, 6, 9, and 12 months. For the purposes of these analyses, clinically meaningful change in MIDAS total score was defined as a 5-point decrease, per previous findings [21], and the proportion of patients with a 50% or greater reduction in MIDAS score was also assessed.

Patients could voluntarily withdraw from the study or could be removed from the study at the discretion of the investigator or sponsor at any time, with the primary reason for discontinuation noted.

### Statistical methods

All analyses were performed on the MIDAS population, which included all patients with any MIDAS assessment post-baseline; the exception was the analysis of “interference with normal activities” during the course of each attack, which included all patients who took study medication and reported interference values at each time point.

MIDAS total score was calculated as the sum of the answers to the 5 questions on the MIDAS questionnaire. Scores were used to assign MIDAS grades as follows: Grade 1: 0–5 = no or little disability; Grade II: 6–10 = mild disability; Grade III: 11–20 = moderate disability; Grade IV: ≥21 = severe disability [18]. Total lost productive days were calculated as the sum of MIDAS question 1 and question 2. A sensitivity analysis was performed assessing total lost productive days in patients with total lost workplace productive days at baseline >0 (sensitivity analysis population), thus excluding patients who had no productive days to gain.

The impact of informative discontinuation on overall treatment response was assessed by comparing patients with a maximum of 1, 2, 3, and 4 quarters of available data versus data for all patients.

Least squares mean MIDAS score changes were modeled using a mixed model repeated measures analysis that included terms for treatment, visit, subgroup, treatment-by-visit, subgroup-by-treatment; subgroup-by-visit; and subgroup-by-treatment-by-visit. Statistical analyses were performed using SAS Version 9.4 or higher (SAS Institute, Cary, North Carolina).

## Results

### Patient characteristics and disposition

Baseline demographic and disease characteristics are shown in Table 1. In the overall MIDAS population, the average age was 43.2 years (range 18 to 79 years) and 85% of patients were women. The majority of the patients were white (77.6%) and 49.1% of patients had ≥2 cardiovascular risk factors. Twenty-two percent of the patients were taking a migraine preventive medication.

**Table 1:** Baseline characteristics by lasmiditan dose for the GLADIATOR MIDAS population

Parameter	Lasmiditan 100 mg (***N*** = 974)	Lasmiditan 200 mg (***N*** = 1063)
Age, mean years (SD)	42.8 (12.3)	43.6 (12.4)
Female, n (%)	828 (85.0)	904 (85.0)
Body mass index, kg/m^2^ (SD)	31.1 (8.2)	31.0 (8.1)
Race		
White, n (%)	744 (76.4)	837 (78.7)
African American, n (%)	193 (19.8)	181 (17.0)
Other, n (%)	37 (3.8)	43 (4.0)
Duration of migraine, mean years (SD)	18.8 (12.8)	18.8 (12.9)
Migraine attacks/month, mean n (SD)^a^	5.2 (1.8)	5.2 (1.8)
Migraine with aura^b,c^, n (%)	356 (36.6)	366 (34.5)
Migraine preventive medication use, n (%)	214 (22.0)	234 (22.0)
Patients with CV risk factors^b,d^, n (%)		
≥ 1	814 (83.6)	852 (80.2)
≥ 2	482 (49.5)	519 (48.8)
MIDAS questionnaire		
MIDAS total score^e^, mean (IQR)	29.4 (15, 36)	28.9 (15, 35)
Headache days in past 3 months, mean (IQR)^f^	15.5 (8, 20)	15.5 (8, 20)
Severity of headache pain^g^, mean (IQR)	7.4 (7, 8)	7.3 (6, 8)

Abbreviations: *CAD* coronary artery disease; *CV* cardiovascular; *IQR* interquartile range; *MIDAS* Migraine Disability Assessment; *SD* standard deviation

^a^Based on response to the question in migraine history section of the case report form “Frequency of migraine attacks (average) during the last three months.” ^b^Data from parent study. ^c^Fulfilling ICHD criteria 1.2.1 for migraine with typical aura. ^d^Hypertension, hypercholesterolemia, smoking, obesity, diabetes mellitus, family history of CAD, men over 40 years of age, and postmenopausal women. ^e^Calculated as the sum of the answers to the 5 questions on the MIDAS questionnaire (0–5 = little or no disability; 6–10 = mild disability; 11–20 = moderate disability; ≥21 = severe disability; >40 = very severe disability). ^f^“On how many days in the past 3 months did you have a headache? (If a headache lasted more than 1 day, count each day)”. ^g^“On a scale of 0 to 10, on average how painful were these headaches? (where 0 = no pain at all, and 10 = pain as bad as it can be)”

Of 2116 patients randomized, 1978 patients received at least 1 dose of lasmiditan (safety population), and 19,058 total migraine attacks were treated. The median time on study was 288 days (IQR, 98–363 days, *n* = 1834). As of the data cut-off for this interim analysis, 814 (41.2% of the safety population) patients had completed the 12 months of study; 141 (7.1%) patients were still receiving treatment; and 1023 (51.7%) patients had discontinued. The most common reason for discontinuation was patient request (21.8%), followed by adverse event (12.8%) and lost to follow-up (9.2%). Safety and tolerability data, as well as data on overall efficacy have been reported previously [14].

### Migraine-related disability

**Fig. 1:**
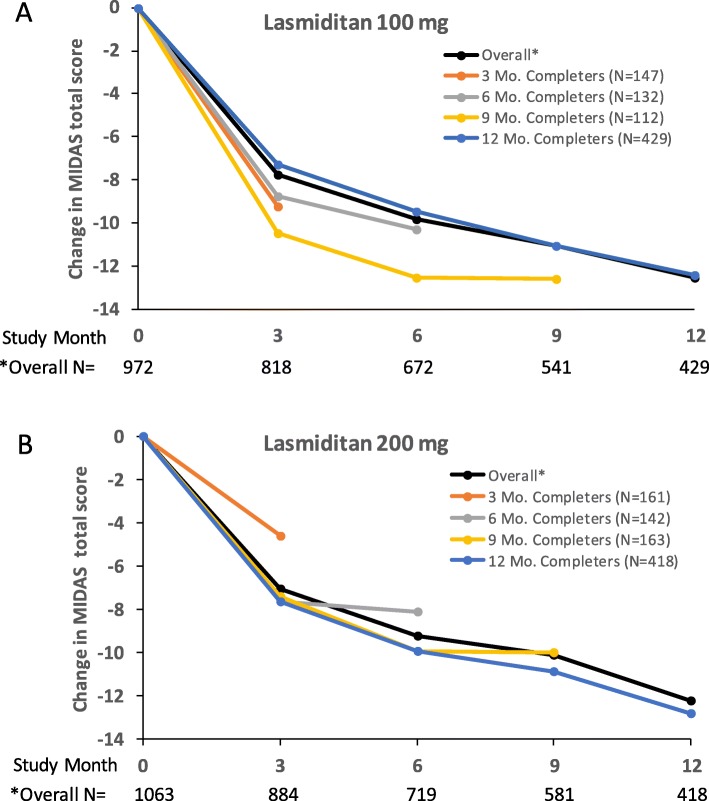
Completer analysis showing MIDAS total score overall and for patients who completed a maximum of 3, 6, 9, or 12 months of study for **a**) Lasmiditan 100 mg and **b**) Lasmiditan 200 mg. Abbreviations: Mo. = month. Note that N within completer subgroups remained nearly constant (within 2 patients) across relevant timepoints

The mean MIDAS total score at baseline was approximately 29, which is in the range of severe migraine disability **(**Table 2**)**. Mean MIDAS total scores decreased significantly from baseline to months 3, 6, 9, and 12 for both the lasmiditan 100 mg and 200 mg treatment groups (Table 2), with no significant differences between the dose groups (all *p* > 0.43). Similarly, no significant differences between lasmiditan dose groups were observed for the mean number of headache days (all *p* > 0.31) and average headache pain over the last 3 months (all *p* > 0.46; Table 2). To examine the effect of loss to follow-up at later time points, we assessed results separately for patients who completed a maximum of 3, 6, 9, and 12 months of study. Findings were similar to those for the whole population in each analysis when assessed by MIDAS total score (Fig. 1) or by headache days or headache pain (Additional file 1: Tables S1 and S2), indicating that the observed response was not broadly impacted by attrition.

**Table 2:** Overall change from baseline in MIDAS total score, headache days, headache pain, and number of treated attacks for patients who completed all 4 quarters of treatment

		Lasmiditan 100 mg (*n* = 974)		Lasmiditan 200 mg (*n* = 1063)	
		Change from BL*		Change from BL*	
		LS mean	% change in LS mean	LS mean	% change in LS mean
MIDAS Total Score	BL, mean^a^	29.4		28.9	
	Month 3	−7.7	−26.3	− 7.0	−24.3
	Month 6	−9.8	− 33.5	− 9.2	− 31.9
	Month 9	− 11.0	− 37.6	− 10.1	− 34.9
	Month 12	−12.5	− 42.6	− 12.2	− 42.2
Headache Days, past 3 months^b, c^	BL, mean	15.5		15.5	
	Month 3	−3.5	− 22.6	− 3.9	− 24.8
	Month 6	−4.5	−28.7	− 4.1	− 26.1
	Month 9	−5.2	− 33.7	− 5.7	− 36.7
	Month 12	−5.7	− 36.8	−6.0	−38.8
Average headache pain intensity^b, d^	BL, mean	7.4		7.3	
	Month 3	−0.4	−5.4	− 0.4	− 5.7
	Month 6	−0.7	−9.1	− 0.7	−9.0
	Month 9	−0.8	−10.4	−0.9	−11.9
	Month 12	−1.1	− 15.1	− 1.2	− 16.6
Number of treated attacks, past 3 months^e^		Lasmiditan 100 mg		Lasmiditan 200 mg	
		N	Mean	N	Mean
	Month 3	326	6.0	320	6.0
	Q2	312	5.0	296	4.9
	Q3	303	4.4	298	4.3
	Q4	336	3.7**	331	3.4**

Abbreviations: *BL* baseline; *LS* mean = least squares mean

^a^Baseline n = 972 for lasmiditan 100 mg. ^b^Not included in MIDAS total score. ^c^“On how many days in the past 3 months did you have a headache? (If a headache lasted more than 1 day, count each day)” ^d^Scale 1–10. ^e^Data shown for patients who completed all 4 quarters. *All changes from baseline significant at *p* < 0.001. **p-values from paired t-tests vs 1st quarter

For the overall population, we also assessed the impact of lasmiditan treatment on absenteeism and presenteeism. For absenteeism, days absent from work or school in the last 3 months decreased from a baseline mean of 3.1 and 3.2 to a mean of 1.9 and 1.6 at 12 months for the lasmiditan 100-mg and 200-mg dose groups, respectively; reductions were statistically significant at all time points (Table 3). For presenteeism, days present at work or school with at least 50% reduced productivity decreased from a baseline mean of 6.3 and 6.2 to a mean of 3.5 and 3.0 at 12 months for the lasmiditan 100-mg and 200-mg groups, respectively; reductions were statistically significant at all time points (Table 3).

**Table 3:** Change from baseline in MIDAS absenteeism (days), presenteeism (days), and total lost productive days

		Lasmiditan 100 mg (n = 972)		Lasmiditan 200 mg (n = 1063)	
		Change from BL*		Change from BL*	
		LS mean*	% change in mean	LS mean	% change in mean
Absenteeism (days)	BL, mean ^a^	3.1		3.2	
	Month 3	−0.6	−20.2	−0.8	−25.1
	Month 6	−0.9	−29.2	− 0.9	−29.5
	Month 9	−1.0	−32.4	−1.0	−30.7
	Month 12	−1.0	−32.4	−1.2	−38.6
Presenteeism (days)	BL, mean ^a^	6.3		6.2	
	Month 3	−1.5	−24.1	−1.4	−21.7
	Month 6	−2.1	−32.4	−1.8	−29.3
	Month 9	−2.3	−36.1	−1.8	−29.6
	Month 12	−2.6	−40.3	−2.3	−37.2
Total lost productive days^b^	BL, mean	9.4		9.4	
	Month 3	−2.2	−23.4	−2.2	− 23.4
	Month 6	−3.0	−31.9	−2.8	−29.8
	Month 9	−3.3	−35.1	−2.8	−29.8
	Month 12	−3.6	−38.3	− 3.5	−37.2

Abbreviations: *BL* baseline; *LS* mean least squares mean

*All changes from baseline significant at *p* < 0.001

^a^Calculated as the sum of MIDAS question 1 (absenteeism from work or school) and question 2 (presenteeism at work or school). ^b^Includes patients with 0 total lost productive days at baseline

We assessed changes in total lost productive work days by summing changes in presenteeism and absenteeism. In the overall population, the mean decrease in lost productive days was significant by 3 months and remained significant through 12 months, with a mean improvement in lost productivity of up to 38% following 1 year of lasmiditan treatment (Table 3). Absenteeism contributed about a third and presenteeism contributed the remaining two-thirds of the total productive days gained. Summing absenteeism and presenteeism gains over a year in the overall population, treatment with lasmiditan was associated with an average annual improvement of up to 12 productive days. Findings were similar in a sensitivity analysis analyzing the subgroup of patients with total lost productive days at baseline > 0 (lasmiditan 100 mg, *n* = 783, lasmiditan 200 mg, *n* = 893), with improvements of up to 42% following 1 year of lasmiditan treatment. A total of 16 productive days were gained on average in the sensitivity analysis population.

**Fig. 2:**
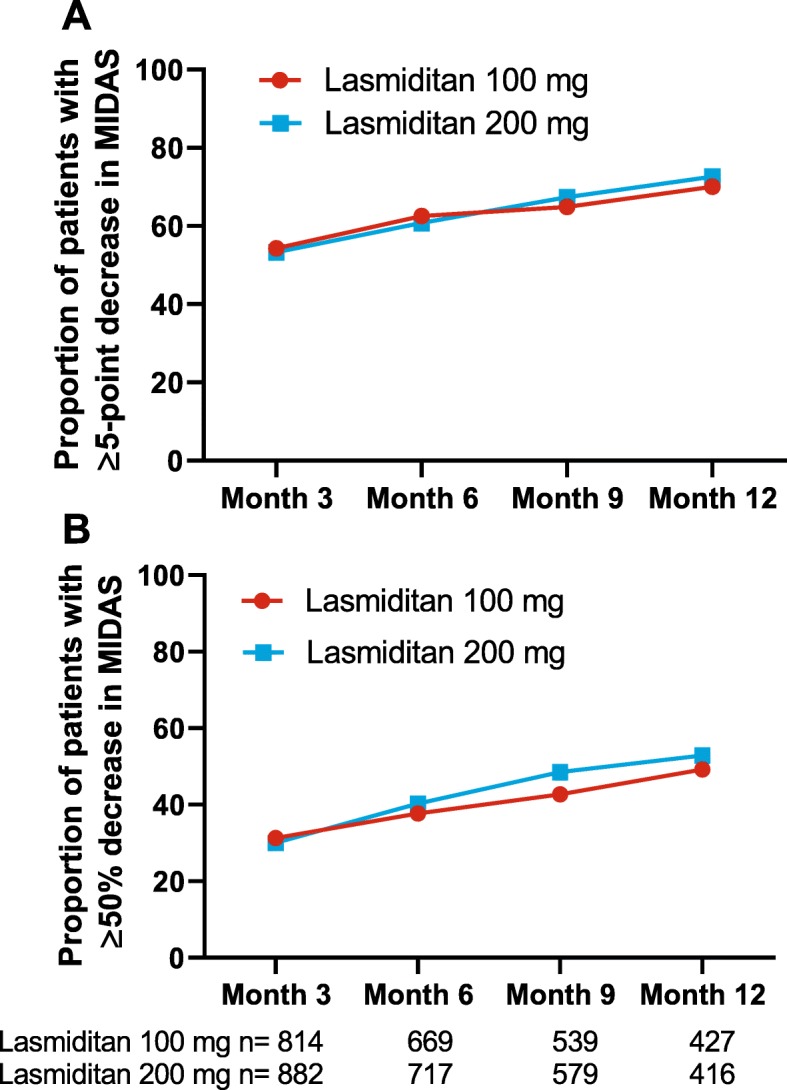
Decreases from baseline in MIDAS total score of (**a**) ≥5 points or (**b**) ≥50%

**Fig. 3:**
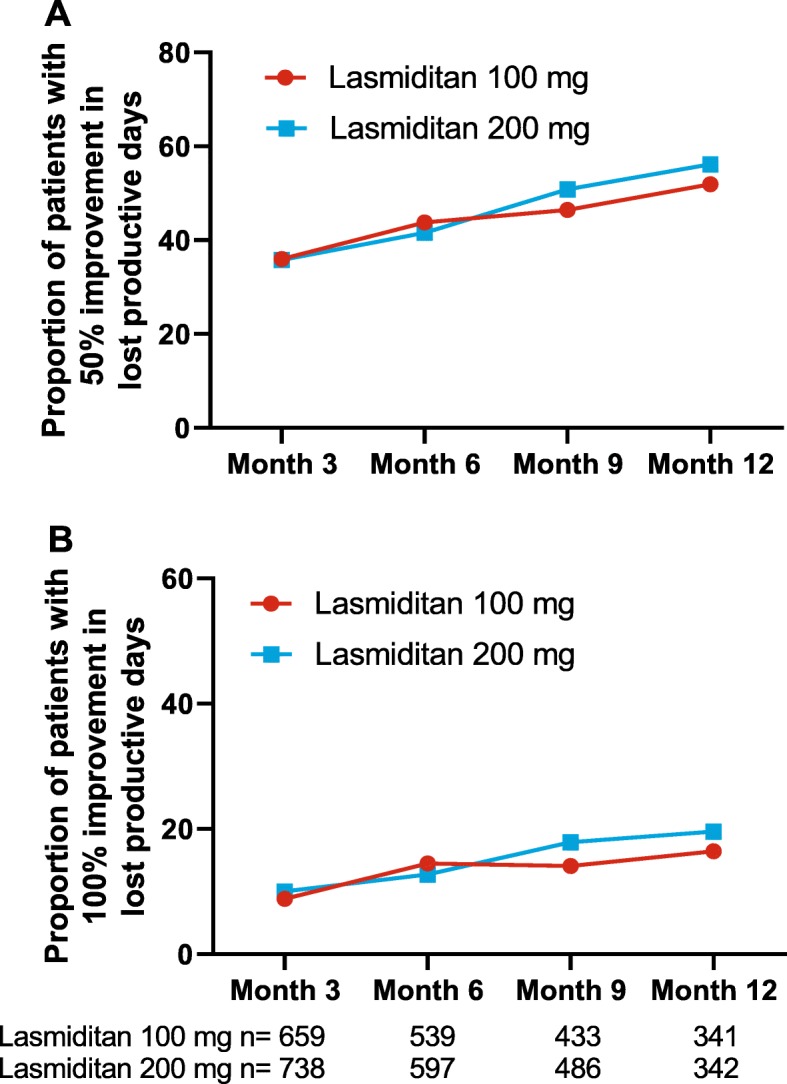
Improvement from baseline in lost productive days of (**a**) ≥50% or (**b**) 100%

Clinically meaningful improvements in MIDAS total score were also assessed over time. For at least a 5-point decrease from baseline, the proportion of responders increased steadily from >53% at 3 months to ≥70% at 12 months. The proportion with at least a 50% decrease in MIDAS total score from baseline increased steadily from ≥30% at 3 months to ≥49% at 12 months (Fig. 2). Responses were generally similar in the 2 dose groups across the different cutoffs. In addition, the proportions of patients with 50% and 100% improvements from baseline in migraine-related total lost workplace productivity were assessed. At 12 months, up to 56% of patients had at least 50% reduction in total lost productive days, and up to 20% had a 100% reduction in total lost productive days due to migraine (Fig. 3).

#### Migraine-related functional impairment over the course of the attack

In addition to assessing long-term migraine disability by the MIDAS instrument, the degree of difficulty in performing activities of daily living was assessed over the first 48 h post-dosing. Across both dose groups, the proportion of patients who reported being able to function normally for the first treated attack was 64.5% by 4 h and 85.7% by 24 h post-lasmiditan dosing (Table 4). Similar improvements were observed from attack 1 through attack 20.

**Table 4:** Observed case analysis of patients free of disability at specified times post-dose for selected attacks

	Time point	Lasmiditan 100 mg^**a**^ n/N (%)	Lasmiditan 200 mg^**a**^ n/N (%)	Overall^**a**^ n/N (%)
Attack 1	Baseline	16/931 (1.7)	26/963 (2.7)	42/1894 (2.2)
	0.5 h	60/797 (7.5)	53/828 (6.4)	113/1625 (7.0)
	1 h	176/814 (21.6)	183/846 (21.6)	359/1660 (21.6)
	2 h	327/847 (38.6)	341/869 (39.2)	668/1716 (38.9)
	4 h	385/585 (65.8)	429/677 (63.4)	814/1262 (64.5)
	24 h	374/432 (86.6)	420/495 (84.8)	794/927 (85.7)
	48 h	365/414 (88.2)	436/504 (86.5)	801/918 (87.3)
Attack 5	Baseline	5/560 (0.9)	5/566 (0.9)	10/1126 (0.9)
	0.5 h	28/477 (5.9)	21/491 (4.3)	49/968 (5.1)
	1 h	80/484 (16.5)	89/510 (17.5)	169/994 (17.0)
	2 h	189/505 (37.4)	194/504 (38.5)	383/1009 (38.0)
	4 h	233/345 (67.5)	249/372 (66.9)	482/717 (67.2)
	24 h	232/271 (85.6)	249/299 (83.3)	481/570 (84.4)
	48 h	243/271 (89.7)	239/286 (83.6)	482/557 (86.5)
Attack 10	Baseline	2/324 (0.6)	4/298 (1.3)	6/622 (1.0)
	0.5 h	11/275 (4.0)	14/262 (5.3)	25/537 (4.7)
	1 h	41/281 (14.6)	48/263 (18.3)	89/544 (16.4)
	2 h	85/285 (29.8)	98/269 (36.4)	183/554 (33.0)
	4 h	109/172 (63.4)	133/192 (69.3)	242/364 (66.5)
	24 h	100/130 (76.9)	116/140 (82.9)	216/270 (80.0)
	48 h	108/121 (89.3)	116/138 (84.1)	224/259 (86.5)
Attack 15	Baseline	1/193 (0.5)	0/174 (0)	1/367 (0.3)
	0.5 h	6/154 (3.9)	6/145 (4.1)	12/299 (4.0)
	1 h	15/157 (9.6)	25/146 (17.1)	40/303 (13.2)
	2 h	43/172 (25.0)	57/156 (36.5)	100/328 (30.5)
	4 h	54/97 (55.7)	65/104 (62.5)	119/201 (59.2)
	24 h	57/69 (82.6)	81/90 (90.0)	138/159 (86.8)
	48 h	63/76 (82.9)	71/85 (83.5)	134/161 (83.2)
Attack 20	Baseline	2/116 (1.7)	0/108 (0)	2/224 (0.9)
	0.5 h	1/98 (1.0)	4/95 (4.2)	5/193 (2.6)
	1 h	12/104 (11.5)	17/92 (18.5)	29/196 (14.8)
	2 h	25/103 (24.3)	36/94 (38.3)	61/197 (31.0)
	4 h	28/52 (53.8)	40/59 (67.8)	68/111 (61.3)
	24 h	33/44 (75.0)	41/51 (80.4)	74/95 (77.9)
	48 h	35/42 (83.3)	43/52 (82.7)	78/94 (83.0)

Abbreviations: n = number of patients in category; N = number of patients with data available

Based on electronic diary question “How much is your migraine interfering with your normal activities.” Response options were “not at all,” “mild interference,” “marked interference,” and “need complete bed rest”. Data shown for “not at all” (no disability). Analysis of “interference with normal activities” during the course of each attack included all patients who took study medication and reported interference values at each time point

### Subgroup analysis by baseline MIDAS Total score severity

Subgroup analysis was performed comparing MIDAS disability <21 vs. ≥21 at baseline. As shown in Table 5, there were notable differences in improvement in MIDAS total score, absenteeism, and presenteeism, and in headache days, between MIDAS severity subgroups at baseline. Decreases from baseline were generally noted in all parameters in both subgroups and with both lasmiditan doses. The decrease from baseline was greater in the subgroup with greater baseline disability based on MIDAS total score (visit-by-subgroup interaction term *p* < 0.001), absenteeism (*p* < 0.001), presenteeism (*p* < 0.001), and headache days (*p* = 0.017). The differences in response between baseline disability subgroups appeared to be greatest for absenteeism and presenteeism both at baseline and during treatment (Table 4).

**Table 5:** Change from baseline in MIDAS response by baseline MIDAS disability severity (<21 vs. ≥21)

		MIDAS total < 21 at baseline				MIDAS total ≥ 21 at baseline			
		Lasmiditan 100 mg (*n* = 418)		Lasmiditan 200 mg (*n* = 465)		Lasmiditan 100 mg (*n* = 554)		Lasmiditan 200 mg(*n* = 598)	
		LS mean	% change in mean	LS mean	% change in mean	LS mean	% change in mean	LS mean	% change in mean
MIDAS Total	Baseline, mean	13.8		13.8		41.1		40.7	
	Month 3	−0.6	−4.2	−1.7**	−12.5	−13.3**	−32.3	− 11.3**	−27.9
	Month 6	−1.9**	−13.9	−2.5**	− 18.3	− 16.1**	− 39.1	− 14.9**	− 36.6
	Month 9	− 2.6**	− 18.9	− 3.9**	−28.0	− 17.8**	− 43.3	− 15.3**	− 37.7
	Month 12	− 4.1**	− 29.7	− 4.2**	− 30.4	− 19.3**	− 47.0	− 19.5**	− 47.8
Absenteesim	Baseline, mean	1.47		1.67		4.37		4.38	
	Month 3	−0.1	−5.4	− 0.4**	−22.2	−1.1**	− 24.0	− 1.2**	− 26.3
	Month 6	− 0.1	−6.1	− 0.2	− 12.0	− 1.5**	−35.2	− 1.6**	− 35.4
	Month 9	− 0.3*	−17.7	− 0.3*	−19.8	− 1.6**	−36.8	−1.5**	− 35.2
	Month 12	− 0.2	−10.2	− 0.4*	− 22.2	− 1.7**	−39.4	− 2.0**	− 45.2
Presenteeism	Baseline, mean	2.77		3.02		9.00		8.70	
	Month 3	−0.02	− 0.7	− 0.2	−7.6	−2.7**	− 29.9	− 2.3**	− 26.0
	Month 6	− 0.1	−4.7	− 0.3	−9.6	−3.6**	− 39.7	− 3.1**	− 35.6
	Month 9	− 0.3	− 10.5	− 0.8**	−24.8	−3.9**	− 43.1	− 2.8**	−31.8
	Month 12	− 0.6*	− 23.1	− 0.6*	− 20.5	− 4.1**	− 45.4	−3.9**	− 44.3
Headache days^a^	Baseline, mean	11.05		10.92		18.89		19.14	
	Month 3	−2.7**	−24.7	− 2.1**	−19.5	−4.1**	− 21.7	−5.2**	−27.4
	Month 6	−2.8**	−25.0	− 2.2**	−20.4	− 5.8**	−30.8	− 5.5**	−28.9
	Month 9	−3.7**	−33.6	− 3.4**	− 31.1	−6.4**	− 34.1	− 7.6**	−39.9
	Month 12	−4.6**	−41.2	−3.3**	−30.0	−6.6**	− 34.9	−8.4**	− 43.9
Average headache pain intensity^a^	Baseline, mean	7.32		7.14		7.46		7.49	
	Month 3	−0.4*	−4.9	− 0.4**	−5.9	− 0.4**	− 5.8	− 0.4**	− 5.6
	Month 6	−0.7**	−10.1	− 0.6**	−8.0	−0.6**	− 8.3	− 0.7**	−9.9
	Month 9	−0.8**	− 10.4	−1.0**	− 14.1	− 0.8**	−10.3	− 0.8**	− 10.0
	Month 12	−1.0**	− 13.8	−1.2**	− 16.7	−1.2**	− 16.1	−1.3**	− 17.0

*p < .05; **p < .001. ^a^Not included in MIDAS total score. Abbreviations: BL = baseline; LS mean = least squares mean; n = number of patients in subgroup

### Response by number of monthly migraine attacks at baseline

**Fig. 4:**
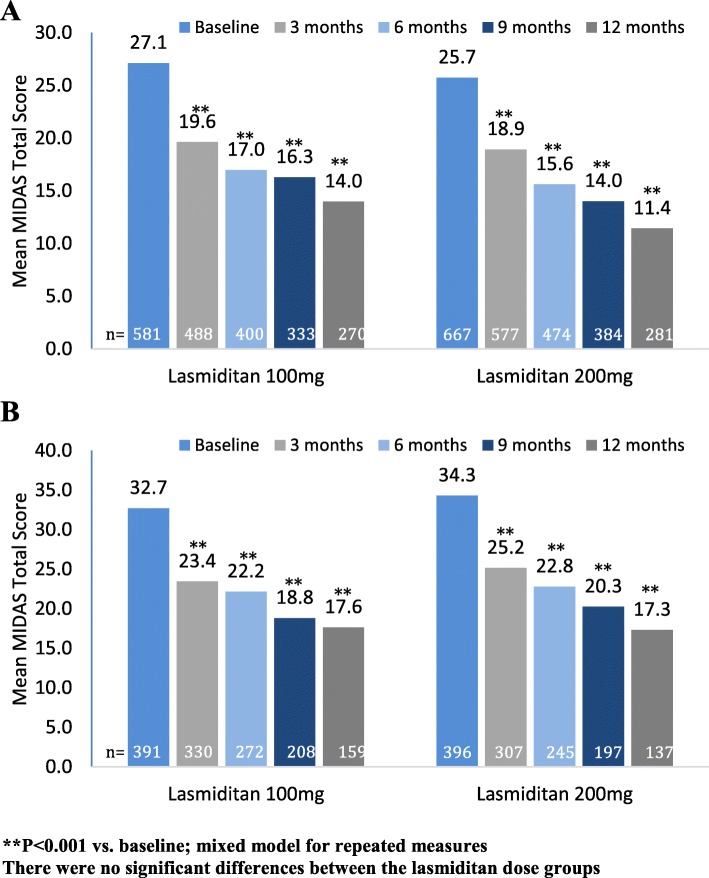
Mean MIDAS score by study month in subgroups defined by monthly migraine attacks at baseline (**a**) ≤5 and (**b**) >5. **P < 0.001 vs. baseline; mixed model for repeated measures. There were no significant differences between the lasmiditan dose groups.

The mean number of monthly migraine attacks at baseline was 5.2 (Table 1). In order to examine whether the frequency of migraine attacks at baseline affected MIDAS reductions, we assessed response for patients with baseline frequency of attacks of ≤5 or >5 per month. At baseline, the mean MIDAS total scores were approximately 26.4 for the ≤5 attacks/month group and 33.5 for the >5 attacks/month group. Mean MIDAS total scores decreased significantly from baseline at all time points for each dose group in both subgroups (all comparisons, *p* < 0.001 vs. baseline; Fig. 4a and b). Similar findings were seen for number of headache days (Additional file 1: Table S3) and for headache pain (except average headache pain for the >5 subgroup, 200-mg dose group at 3 months, *p* = 0.004; Additional file 1: Table S4). Subgroup analysis for number of monthly attacks showed a greater decrease from baseline over time in the >5 than in the ≤5 subgroup (visit-by-subgroup interaction term *p* < 0.001).

### Subgroup analyses by pain-free response at 2 hours

To examine whether pain-free responses at 2 h for the first few treated attacks predicted change in MIDAS with lasmiditan treatment, we categorized participants into two groups, those who achieved pain freedom 2 h postdose and those who did not for the first migraine attack, and for 2 of the first 3 migraine attacks.

**Fig. 5:**
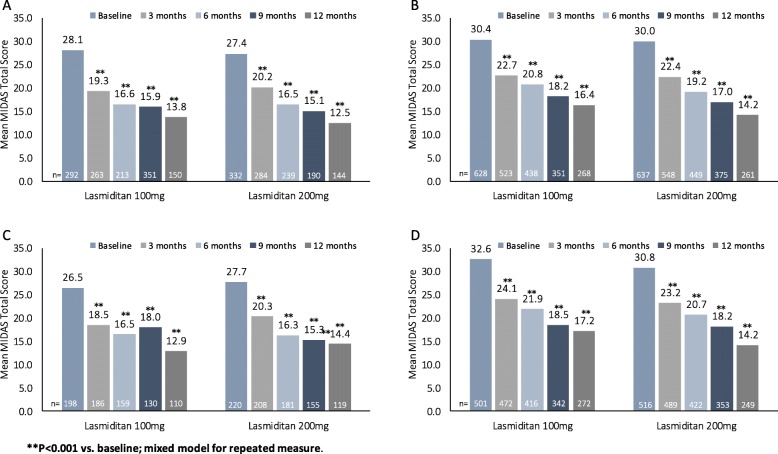
Mean MIDAS Score by pain-free response 2 h post-dose (**a**) yes, first attempt, (**b**) no, first attempt, (**c**) yes, ≥2 of first 3 attempts (**d**) no, ≥2 of first 3 attempts (intent-to-treat population). **P < 0.001 vs. baseline; mixed model for repeated measures There were no significant differences between the lasmiditan doses.

For the lasmiditan 100-mg and 200-mg treatment groups, 31.7% (292/920) and 34.3% (332/969) of patients, respectively, achieved pain-free response on their first treated attack in GLADIATOR. For subgroups based on pain-free response for the first migraine attack, baseline mean MIDAS total scores differed only slightly (approximately 27.8 for pain free at 2 h, 30.2 for not pain free), and MIDAS total scores decreased significantly from baseline at all time points for each dose group in both subgroups (all comparisons, *p* < 0.001 vs. baseline; Fig. 5a and b). Changes in MIDAS total scores over time were similar between the 2 subgroups. The findings were generally similar for these subgroups for number of headache days (Additional file 1: Table S5), and also for headache pain (Additional file 1: Table S6).

For pain-free response at 2 h in at least 2 of the first 3 treatment attempts, 28.3% (198/699) and 29.9% (220/736) of patients treated with lasmiditan 100 mg and 200 mg respectively, achieved positive response. Baseline mean MIDAS values differed somewhat (approximate means 27.1 and 31.7, respectively) between patients with pain-free response in at least 2 of 3 attacks vs those who did not. MIDAS total scores decreased significantly from baseline at all timepoints for each dose group in both subgroups (all comparisons, *p* < 0.001 vs. baseline; Fig. 5c and d). Changes were also significant for number of headache days across dose groups for both subgroups (all comparisons, *p *< 0.001 vs. baseline, Additional file 1: Table S7), with no significant differences between doses. For average headache pain, changes from baseline were significant across dose groups at all timepoints in the not pain free for at least 2 of the first 3 attempts subgroup (all *p* < 0.05, Additional file 1: Table S8); for the subgroup that was pain free at 2 h for at least 2 of the first 3 attempts, average headache pain decreased from baseline for both doses and at all time points. Statistical significance (*p* < 0.05) was achieved at 3 and 12 months for the lasmiditan 100-mg group and at 6, 9, and 12 months for the lasmiditan 200-mg dose group.

## Discussion

Migraine imposes a significant burden on family, work, school, and social life, especially to those under age 55. [3, 5] The MIDAS is a valid and reliable migraine-related disability measure that is strongly correlated with clinical judgement of the severity of migraine and the need for healthcare services [22]. In the interim results from the GLADIATOR study, significant and increasing improvements in MIDAS total score and mean headache days were reported with intermittent use of lasmiditan for up to a year [14]. At baseline, patients had an average MIDAS score of 29, representing a migraine population with severe migraine-related disability. Results from the present study demonstrate that decreases in MIDAS total scores were statistically significant for both lasmiditan doses by 3 months and that those benefits were sustained from 3 through 12 months. Benefits were generally consistent across subgroups assessed by number of monthly attacks at baseline, pain-free response at 2 h on the first attempt, and pain-free response at 2 h on at least 2 of the first 3 attempts. The magnitude of MIDAS reduction was greater in persons with MIDAS scores ≥21 compared to those with baseline scores of <21. Mean MIDAS total scores decreased with increasing duration in the study. In addition, a clinically meaningful response of at least a 5-point decrease from baseline in MIDAS total score [21] was seen in a steadily increasing proportion of patients over time, reaching ≥70% across the 2 lasmiditan dose groups at 12 months.

In this study, MIDAS scores, headache days, and headache severity decreased significantly and progressively during treatment over a follow-up period of up to a year. Only limited data are available for MIDAS changes during treatment with acute medications for migraine. A study of sumatriptan plus naproxen showed a baseline MIDAS score of 28.7 and a follow-up MIDAS of 22.6 after 3 months of treatment. Similarly, a naproxen-only group showed a baseline of 27.9 and a follow-up MIDAS score of 24.1 after 3 months of treatment [23]. In a study of 62 Japanese migraine patients treated with triptans for 17 months, MIDAS score was 27.2 at baseline and 15.5 at endpoint (*p* = .0002); however, headache days were similar at baseline (27.5) and at the end of study (23.4, *p* = NS) [24]. Long-term safety studies provide some insight into the relative stability of migraine disease during treatment with triptans. For example, in a study of almotriptan for 12 months, the average number of headaches treated in month 1 was 2.39 and in month 12 was 2.37 [25]. In a one-year study of zolmitriptan nasal spray, the mean number of headaches treated per person across 90-day treatment periods ranged from 10.0 to 10.6 [26]. For frovatriptan, migraine days were 10.8 at baseline and 9.6 after 6 months of treatment [27]. Finally, in a rizatriptan study, mean monthly headache rate was ~ 4.2 during months 1–3 and ~ 3.3 during months 10–12 [28]. Thus while mean migraine-related disability and headache days decreased for patients receiving lasmiditan in the present study, treatment with triptans results in variable effects on migraine disability and a relatively stable number of migraine headache days at a population level. Migraine attack frequency may increase or decrease over time [29]. The decrease in headache days with lasmiditan treatment may represent a preventive effect of lasmiditan or may be a result of the natural history of the disease or some other unknown effect; in the absence of a placebo control arm in this long-term safety study, we cannot distinguish these possibilities.

In GLADIATOR, absenteeism decreased for both lasmiditan doses at all time points during treatment for up to a year. Additionally, presenteeism (continuing to work despite reduced effectiveness) also decreased significantly for both lasmiditan doses at all time points. These results indicate that lasmiditan may reduce both complete loss of work days and days with impaired functioning.

Workplace productivity improvements following successful migraine treatment can have a substantial impact on lives of people with migraine. Total workplace productivity gains can be estimated by summing presenteeism and absenteeism gains [30–32]. Treatment with lasmiditan was associated with an average reduction in lost productivity of up to 12 days per year, which equates to approximately $2700 per person per year based on an average US hourly wage of ~$28 [33].

In a comparison of those with higher versus lower disability (baseline total MIDAS total score moderate <21 vs. severe ≥21), both subgroups showed improvements in total MIDAS score, headache days, headache pain, absenteeism, and presenteeism. However, a greater response to lasmiditan was generally seen for patients with MIDAS disability scores rated as severe at baseline. Notably, the MIDAS total score directly includes measures of absenteeism and presenteeism scores (along with lost time in household work as well as family, social, and leisure activities), but not the scores for headache days and headache pain. This is likely to have impacted the results for absenteeism and presenteeism in the ≥21 baseline MIDAS total score subgroup since the markedly higher (approaching 3-fold) baseline scores for absenteeism and presenteeism allow greater range for improvement due to treatment effects and to unrelated regression to the mean. These results show that patients treated with lasmiditan with either moderate or severe migraine-related disability on average appeared to have an improvement in their disability, with greater improvement in the severely disabled patients.

One factor that could contribute to the decrement in disability over time is a decrease in the frequency of attacks. In this study, headache days decreased significantly and progressively during treatment for up to a year; the mean number of migraine attacks treated with lasmiditan per patient per quarter decreased over time from 6.0 to 3.7 for lasmiditan 100 mg and 6.0 to 3.4 for lasmiditan 200 mg dose [14]. Another factor that could contribute to the overall improvements in disability could be reduced disability associated with each individual attack. In GLADIATOR, the proportion of patients with no disability increased over the course of the treated attack, with 64.5% reporting no disability 4 h post-treatment and 85.7% reporting no disability 24 h post-treatment. Reduced disability during each attack could also contribute to reduced presenteeism and absenteeism over time. Thus, both decrease in the frequency of attack and reduction in disability per attack likely contributed to the overall reduction in MIDAS over time.

In longitudinal analyses such as these, it is important to evaluate the potential impact of loss to follow-up. If patients with a poorer treatment response are more likely to drop out, that could contribute to the decrement in disability over time. To assess this issue, we compared results in completers with those in patients who dropped out, an approach in line with the guidance from the National Academy of Science white paper on missing data in clinical trials [34]. Analyses of patients with a maximum of 3, 6, 9, and 12 months of data showed similar results, suggesting that selective attrition did not have a major impact on our conclusions regarding reductions in disability.

Limitations of the present study include that it was an open-label design, and thus lacked a placebo control group for comparison. As a consequence, we cannot control for placebo effects or for regression to the mean. In addition, there was a somewhat higher rate of patient dropout (51.7%) than seen in a 12-month study of telcagepant (42.4%) versus rizatriptan (35.1%) [28], as well as earlier long-term studies of zolmitriptan [35] and almotriptan [25]. Discontinuation over time could result in retention of a sample for whom lasmiditan has greater efficacy, potentially resulting in accumulating reductions in MIDAS scores. However, analyses of patients with a maximum of 1, 2, 3, and 4 available quarters of data showed similar results, suggesting that attrition was not an obvious contributor to our results. The validity of the 3-month MIDAS questionnaire has been evaluated against a daily diary. Although individuals may underestimate the severity of individual attacks or selectively recall more severe attacks, overall MIDAS scores were highly correlated with results from a daily diary [19]. Additionally, the MIDAS questionnaire was developed with a 3-month recall interval to balance the accuracy of self-reported information with a long enough recall interval to capture a representative amount of headache experience [18]. A limitation of the analysis of attack-related disability was that data were not available for all patients at all time points, as between 2 and 24 h after the first dose of study drug, patients were allowed to take a second dose of study drug for rescue or recurrence of headache, after which disability data relative to first dose were no longer collected. Finally, as part of the overall study design, patients were randomized to receive lasmiditan 100 mg or 200 mg and dose adjustment was not permitted, perhaps contributing to discontinuations for both efficacy and tolerability reasons.

In conclusion, the interim efficacy results presented here show progressive and clinically meaningful reductions in migraine-related disability during long-term dosing with lasmiditan. Patients with baseline severe versus moderate migraine-related disability showed greater reductions in disability over time. Response to lasmiditan per the MIDAS score was consistent across subgroups of baseline headache frequency, and of response to lasmiditan for initial treated attacks.

## Supplementary information

**Additional file 1:** The Migraine Disability Assessment (MIDAS) Questionnaire [1–3]. **Table S1.** Change in mean days with headache over the last 3 months by completer status. **Table S2.** Change in mean headache pain intensity over the last 3 months by completer status. **Table S3.** Change in mean days with headache over the last 3 months by baseline number of migraine attacks. **Table S4.** Change in mean headache pain intensity during the past 3 months by baseline number of migraine attacks. **Table S5.** Change in mean days with headache over the past 3 months by pain-free response at 2 h post-dose for the first treated attack. **Table S6.** Change in mean headache pain intensity over the last 3 months by pain-free response at 2 h post-dose for the first treated attack. **Table S7.** Change in mean days with headache over the past 3 months by pain-free response at 2 h on ≥2 of first 3 attempts yes/no. **Table S8** Change in mean headache pain intensity over the last 3 months by pain-free response at 2 h on ≥2 of first 3 attempts yes/no.

## Data Availability

Lilly provides access to all individual participant data collected during the trial, after anonymization, with the exception of pharmacokinetic or genetic data. Data are available to request 6 months after the indication studied has been approved in the US and EU and after primary publication acceptance, whichever is later. No expiration date of data requests is currently set once data are made available. Access is provided after a proposal has been approved by an independent review committee identified for this purpose and after receipt of a signed data sharing agreement. Data and documents, including the study protocol, statistical analysis plan, clinical study report, and blank or annotated case report forms, will be provided in a secure data sharing environment for up to 2 years per proposal. For details on submitting a request, see the instructions provided at www.clinicalstudydatarequest.com.
