# Supplementary material for: Trajectory of migraine-related disability following long-term treatment with lasmiditan: results of the GLADIATOR study
Source: J Headache Pain. 2020 Feb 24;21(1):20. doi: 10.1186/s10194-020-01088-4 (PMC7041198; doi:10.1186/s10194-020-01088-4)
Supplement: Supplementary file 1 — Additional file 1: The Migraine Disability Assessment (MIDAS) Questionnaire [1–3]. Table S1. Change in mean days with headache over the last 3 months by completer status. Table S2. Change in mean headache pain intensity over the last 3 months by completer status. Table S3. Change in mean days with headache over the last 3 months by baseline number of migraine attacks. Table S4. Change in mean headache pain intensity during the past 3 months by baseline number of migraine attacks. Table S5. Change in mean days with headache over the past 3 months by pain-free response at 2 h post-dose for the first treated attack. Table S6. Change in mean headache pain intensity over the last 3 months by pain-free response at 2 h post-dose for the first treated attack. Table S7. Change in mean days with headache over the past 3 months by pain-free response at 2 h on ≥2 of first 3 attempts yes/no. Table S8 Change in mean headache pain intensity over the last 3 months by pain-free response at 2 h on ≥2 of first 3 attempts yes/no. [file 10194_2020_1088_MOESM1_ESM.docx]

**Supplementary Files**

**The Migraine Disability Assessment (MIDAS) Questionnaire** (1-3)

- On how many days in the past 3 months did you miss work or school because of your headaches?
- How many days in the past 3 months was your productivity at work or school reduced by half or more because of your headaches? (Do not include days you counted in question 1 where you missed work or school)
- On how many days in the past 3 months did you not do household work (such as housework, home repairs and maintenance, shopping, caring for children and relatives) because of your headaches?
- How many days in the past 3 months was your productivity in household work reduced by half of more because of your headaches? (Do not include days you counted in question 3 where you did not do household work)
- On how many days in the past 3 months did you miss family, social or leisure activities because of your headaches?

The MIDAS total score is the sum of the 5 questions above, with 0-5 = little or no disability, 6‑10 = mild disability, 11-20 = moderate disability, and ≥21 = severe disability.

Two additional questions assess the number of headache days and severity of headaches:

- On how many days in the past 3 months did you have a headache? (If a headache lasted more than 1 day, count each day)
- On a scale of 0 to 10, on average how painful were these headaches? (where 0 = no pain at all, and 10 = pain as bad as it can be)

**References**

1. Stewart WF, Lipton RB, Kolodner K, et al. Reliability of the migraine disability assessment score in a population-based sample of headache sufferers. Cephalalgia. 1999;19:107-14.
2. Stewart WF, Lipton RB, Whyte J, et al. An international study to assess reliability of the Migraine Disability Assessment (MIDAS) score. Neurology. 1999;53 988-94.
3. Stewart WF, Lipton RB, Dowson AJ, et al. Development and testing of the Migraine Disability Assessment (MIDAS) Questionnaire to assess headache-related disability. Neurology. 2001;56:S20-S28.

**Supplemental Table 1. Change in mean days with headache over the last 3 months by completer status**

|  | **Overall** | | | | | **3 month completers** | | | | **6 month completers** | | | | | | | |  |  |  |
| --- | --- | --- | --- | --- | --- | --- | --- | --- | --- | --- | --- | --- | --- | --- | --- | --- | --- | --- | --- | --- |
|  | Lasmiditan 100 mg | | Lasmiditan 200 mg | | | Lasmiditan 100 mg | | Lasmiditan 200 mg | | Lasmiditan 100 mg | | | | | Lasmiditan 200 mg | | |  |  |  |
|  | Mean (SD) | LS mean change | Mean (SD) | | LS mean change | Mean (SD) | LS mean change | Mean (SD) | LS mean change | Mean (SD) | LS mean change | | | | Mean (SD) | LS mean change | |  |  |  |
| Baseline | 15.5 (11.0) | -- | 15.5 (11.5) | | -- | 15.9 (11.8) | -- | 16.8 (12.0) | -- | 17.5 (12.4) | -- | | | | 14.9 (11.0) | -- | |  |  |  |
| Month 3 | 11.8 (11.1) | -3.5 | 11.3 (10.0) | | -3.9 | 12.8 (13.3) | -3.2 | 11.7 (11.4) | -5.1 | 14.3 (14.2) | -3.2 | | | | 11.3 (8.2) | -3.7 | |  |  |  |
| Month 6 | 10.6 (10.1) | -4.5 | 10.9 (11.9) | | -4.1 | -- | -- | -- | -- | 11.4 (11.7) | -6.1 | | | | 12.9 (16.7) | -2.0 | |  |  |  |
| Month 9 | 9.5 (8.7) | -5.2 | 9.0 (9.6) | | -5.7 | -- | -- | -- | -- | -- | -- | | | | -- | -- | |  |  |  |
| Month 12 | 8.8 (10.2) | -5.7 | 8.2 (9.5) | | -6.0 | -- | -- | -- | -- | -- | -- | | | | -- | -- | |  |  |  |
|  | | | | | | | | | | | | | | | | | |  |  |  |
|  | **9 month completers** | | | | | **12 month completers** | | | |  | | | | | | | | | |  |
|  | Lasmiditan 100 mg | | Lasmiditan 200 mg | | | Lasmiditan 100 mg | | Lasmiditan 200 mg | |  | |  |  |  |  |  |  |  |  |  |
|  | Mean (SD) | LS mean change | Mean (SD) | LS mean change | | Mean (SD) | LS mean change | Mean (SD) | LS mean change |  | | |  |  | | |  | |  | |
| Baseline | 17.4 (12.2) | -- | 15.9 (11.8) | -- | | 14.3 (9.5) | -- | 14.5  (10.7) | -- |  | | |  |  | | |  | |  | |
| Month 3 | 11.9 (10.3) | -5.5 | 12.8 (11.3) | -3.1 | | 10.6 (9.0) | -3.7 | 10.6 (9.4) | -3.9 |  | | |  |  | | |  | |  | |
| Month 6 | 11.5 (11.0) | -5.9 | 11.5 (11.0) | -4.3 | | 10.2 (9.3) | -4.1 | 10.0 (10.1) | -4.4 |  | | |  |  | | |  | |  | |
| Month 9 | 10.4 (10.2) | -7.0 | 10.3 (10.5) | -5.6 | | 9.2  (8.2) | -5.0 | 8.5 (9.2) | -5.9 |  | | |  |  | | |  | |  | |
| Month 12 | -- | -- | -- | -- | | 8.8 (10.2) | -5.5 | 8.2 (9.5) | -6.3 |  | | |  |  | | |  | |  | |

**Supplemental Table 2. Change in mean headache pain intensity over the last 3 months by completer status**

|  | **Overall** | | | | **3 month completers** | | | | **6 month completers** | | | |
| --- | --- | --- | --- | --- | --- | --- | --- | --- | --- | --- | --- | --- |
|  | Lasmiditan 100 mg | | Lasmiditan 200 mg | | Lasmiditan 100 mg | | Lasmiditan 200 mg | | Lasmiditan 100 mg | | Lasmiditan 200 mg | |
|  | Mean (SD) | LS mean change | Mean (SD) | LS mean change | Mean (SD) | LS mean change | Mean (SD) | LS mean change | Mean (SD) | LS mean change | Mean (SD) | LS mean change |
| Baseline | 7.4 (1.7) | -- | 7.3 (1.6) | -- | 7.4 (1.5) | -- | 7.3 (1.6) | -- | 7.3 (1.7) | -- | 7.4 (1.5) | -- |
| Month 3 | 7.0 (2.0) | -0.4 | 6.9 (2.0) | -0.4 | 6.8 (2.1) | -0.6 | 7.0 (2.1) | -0.3 | 6.7 (2.1) | -0.6 | 6.9 (2.0) | -0.5 |
| Month 6 | 6.7 (2.3) | -0.7 | 6.7 (2.2) | -0.7 | -- | -- | -- | -- | 6.2 (2.7) | -1.1 | 6.4 (2.5) | -1.0 |
| Month 9 | 6.7 (2.3) | -0.8 | 6.5 (2.4) | -0.9 | -- | -- | -- | -- | -- | -- | -- | -- |
| Month 12 | 6.4 (2.5) | -1.1 | 6.2 (2.6) | -1.2 | -- | -- | -- | -- | -- | -- | -- | -- |
|  | | | | | | | | | | | | |
|  | **9 month completers** | | | | **12 month completers** | | | |  | | | |
|  | Lasmiditan 100 mg | | Lasmiditan 200 mg | | Lasmiditan 100 mg | | Lasmiditan 200 mg | |  | |  | |
|  | Mean (SD) | LS mean change | Mean (SD) | LS mean change | Mean (SD) | LS mean change | Mean (SD) | LS mean change |  |  |  |  |
| Baseline | 7.4 (1.9) | -- | 7.2 (1.8) | -- | 7.4 (1.7) | -- | 7.4 (1.5) | -- |  |  |  |  |
| Month 3 | 7.0 (2.1) | -0.4 | 6.7 (2.1) | -0.5 | 7.1 (1.8) | -0.3 | 7.0 (1.8) | -0.4 |  |  |  |  |
| Month 6 | 6.6 (2.5) | -0.8 | 6.6 (2.1) | -0.6 | 6.9 (2.1) | -0.5 | 6.8 (2.2) | -0.6 |  |  |  |  |
| Month 9 | 6.2 (2.8) | -1.2 | 6.4 (2.3) | -0.8 | 6.8 (2.2) | -0.6 | 6.5 (2.4) | -0.9 |  |  |  |  |
| Month 12 | -- | -- | -- | -- | 6.4 (2.5) | -1.0 | 6.2 (2.6) | -1.2 |  |  |  |  |

**Supplemental Table 3. Change in mean days with headache over the last 3 months by baseline number of migraine attacks**

|  | **Mean migraine attacks per month ≤5** | | | | **Mean migraine attacks per month >5** | | | |
| --- | --- | --- | --- | --- | --- | --- | --- | --- |
|  | Lasmiditan 100 mg | | Lasmiditan 200 mg | | Lasmiditan 100 mg | | Lasmiditan 200 mg | |
|  | Mean (SD) | LS mean change | Mean (SD) | LS mean change | Mean (SD) | LS mean change | Mean (SD) | LS mean change |
| Baseline | 13.9 (10.4) |  | 13.4 (9.3) |  | 18.0 (11.3) |  | 19.2 (13.7) |  |
| Month 3 | 11.0 (10.8) | -2.5 | 10.3 (8.8) | -3.0 | 12.9 (11.4) | -5.0 | 13.2 (11.7) | -5.5 |
| Month 6 | 9.6 (9.1) | -3.8 | 9.5 (9.0) | -3.5 | 12.2 (11.3) | -5.5 | 13.6 (15.8) | -5.1 |
| Month 9 | 9.0 (7.7) | -4.1 | 8.4 (8.3) | -4.7 | 10.3 (10.0) | -7.0 | 12.2 (11.6) | -7.6 |
| Month 12 | 8.0 (9.4) | -4.8 | 7.8 (8.5) | -4.8 | 10.1 (11.3) | -6.9 | 9.1 (11.3) | -8.4 |

LS mean change from baseline, all *p*<.001.

|  | **Mean migraine attacks per month ≤5** | | | | **Mean migraine attacks per month >5** | | | |
| --- | --- | --- | --- | --- | --- | --- | --- | --- |
|  | Lasmiditan 100 mg | | Lasmiditan 200 mg | | Lasmiditan 100 mg | | Lasmiditan 200 mg | |
|  | Mean (SD) | LS mean change | Mean (SD) | LS mean change | Mean (SD) | LS mean change | Mean (SD) | LS mean change |
| Baseline | 7.3 (1.7) |  | 7.3 (1.6) |  | 7.5 (1.5) |  | 7.4 (1.6) |  |
| Month 3 | 7.0 (2.0) | -0.3 | 6.9 (2.0) | -0.5 | 6.9 (2.0) | -0.6 | 7.1 (1.9) | -0.3 |
| Month 6 | 6.6 (2.5) | -0.6 | 6.7 (2.2) | -0.6 | 6.9 (2.2) | -0.8 | 6.6 (2.1) | -0.7 |
| Month 9 | 6.7 (2.4) | -0.6 | 6.5 (2.4) | -0.9 | 6.6 (2.2) | -1.0 | 6.6 (2.2) | -0.9 |
| Month 12 | 6.5 (2.5) | -0.9 | 6.1 (2.7) | -1.3 | 6.3 (2.6) | -1.4 | 6.3 (2.4) | -1.2 |

**Supplemental Table 4. Change in mean headache pain intensity during the past 3 months by baseline number of migraine attacks**

LS mean change from baseline, all *p*<0.001 except lasmiditan 200 mg at month 3 for >5 headaches/month, *p*=.004.

**Supplemental Table 5. Change in mean days with headache over the past 3 months by pain-free response at 2 hours post-dose for the first treated attack**

|  | **2 hour pain-free on first attempt** | | | | **Not 2 hour pain-free on first attempt** | | | |
| --- | --- | --- | --- | --- | --- | --- | --- | --- |
|  | Lasmiditan 100 mg | | Lasmiditan 200 mg | | Lasmiditan 100 mg | | Lasmiditan 200 mg | |
|  | Mean (SD) | LS mean change | Mean (SD) | LS mean change | Mean (SD) | LS mean change | Mean (SD) | LS mean change |
| Baseline | 15.5 (9.8) |  | 15.2 (11.4) |  | 15.9 (11.7) |  | 16.0 (11.5) |  |
| Month 3 | 11.7 (9.7) | -3.6 | 10.7 (9.2) | -4.3 | 12.1 (11.8) | -3.5 | 12.1 (10.6) | -3.4 |
| Month 6 | 10.5 (10.1) | -5.0 | 9.8 (10.6) | -4.6 | 11.0 (10.2) | -4.2 | 11.6 (12.7) | -3.7 |
| Month 9 | 9.8 (8.3) | -5.5 | 8.4 (8.8) | -5.8 | 9.3 (8.9) | -5.2 | 9.4 (10.1) | -5.6 |
| Month 12 | 9.2 (10.7) | -6.3 | 7.8 (8.1) | -6.0 | 8.5 (10.1) | -5.4 | 8.5 (10.3) | -5.9 |

LS mean change from baseline, all *p*<0.001 from baseline

**Supplemental Table 6. Change in mean headache pain intensity over the last 3 months by pain-free at 2 hours post dose for the first treated attack**

|  | **Pain-free at 2 hours on first attempt** | | | | **Not pain-free at 2 hours on first attempt** | | | |
| --- | --- | --- | --- | --- | --- | --- | --- | --- |
|  | Lasmiditan 100 mg | | Lasmiditan 200 mg | | Lasmiditan 100 mg | | Lasmiditan 200 mg | |
|  | Mean (SD) | LS mean change | Mean (SD) | LS mean change | Mean (SD) | LS mean change | Mean (SD) | LS mean change |
| Baseline | 7.2 (1.6) |  | 7.3 (1.5) |  | 7.5 (1.7) |  | 7.3 (1.6) |  |
| Month 3 | 6.9 (2.0) | -0.4 | 7.1 (1.7) | -0.2 | 7.1 (1.9) | -0.4 | 7.0 (1.9) | -0.4 |
| Month 6 | 6.7 (2.3) | -0.6 | 6.8 (2.1) | -0.5 | 6.8 (2.3) | -0.6 | 6.7 (2.2) | -0.6 |
| Month 9 | 6.8 (2.2) | -0.6 | 6.6 (2.3) | -0.8 | 6.7 (2.3) | -0.8 | 6.5 (2.4) | -0.8 |
| Month 12 | 6.6 (2.4) | -0.9 | 6.4 (2.6) | -1.1 | 6.4 (2.6) | -1.2 | 6.2 (2.6) | -1.2 |

LS mean change from baseline, all *p* <0.001 except 200 mg dose at 3 months for pain-free group, *p*=0.059

**Supplemental Table 7. Change in mean days with headache over the past 3 months by pain-free response at 2 hours on ≥2 of first 3 attempts yes/no**

|  | **Pain-free at 2 hours on ≥2 of first 3 attempts** | | | | **Not pain-free at 2 hours on ≥2 of first 3 attempts** | | | |
| --- | --- | --- | --- | --- | --- | --- | --- | --- |
|  | Lasmiditan 100 mg | | Lasmiditan 200 mg | | Lasmiditan 100 mg | | Lasmiditan 200 mg | |
|  | Mean (SD) | LS mean change | Mean (SD) | LS mean change | Mean (SD) | LS mean change | Mean (SD) | LS mean change |
| Baseline | 15.9 (10.7) |  | 15.3 (11.0) |  | 16.4 (11.6) |  | 16.2 (11.8) |  |
| Month 3 | 12.3 (10.0) | -3.7 | 10.8 (9.6) | -4.4 | 12.6 (12.0) | -3.8 | 12.7 (10.6) | -3.5 |
| Month 6 | 10.6 (8.8) | -5.4 | 9.5 (8.8) | -5.3 | 11.4 (11.1) | -4.4 | 12.2 (13.3) | -3.6 |
| Month 9 | 9.7 (8.3) | -5.7 | 8.1 (9.0) | -6.3 | 9.9 (9.2) | -5.4 | 9.9 (10.3) | -5.6 |
| Month 12 | 7.8 (6.0) | -7.3 | 7.7 (9.4) | -6.1 | 9.4 (12.0) | -5.4 | 9.0 (10.1) | -6.0 |

LS mean change from baseline, all *p*<0.001 from baseline

**Supplemental Table 8. Change in mean headache pain intensity over the last 3 months by pain free at 2 hours on ≥2 of first 3 attempts yes/no**

|  | **Pain-free at 2 hours on ≥2 of first 3 attempts** | | | | **Not pain-free at 2 hours on ≥2 of first 3 attempts** | | | |
| --- | --- | --- | --- | --- | --- | --- | --- | --- |
|  | Lasmiditan 100 mg | | Lasmiditan 200 mg | | Lasmiditan 100 mg | | Lasmiditan 200 mg | |
|  | Mean (SD) | LS mean change | Mean (SD) | LS mean change | Mean (SD) | LS mean change | Mean (SD) | LS mean change |
| Baseline | 7.2 (1.7) |  | 7.2 (1.4) |  | 7.4 (1.7) |  | 7.3 (1.7) |  |
| Month 3 | 6.8 (2.0) | -0.4* | 7.2 (1.7) | -0.1 | 7.2 (1.7) | -0.2* | 7.1 (1.7) | -0.2* |
| Month 6 | 7.1 (1.9) | -0.2 | 6.8 (2.2) | -0.5* | 6.8 (2.2) | -0.5** | 6.9 (2.0) | -0.5** |
| Month 9 | 7.1 (2.1) | -0.3 | 6.6 (2.3) | -0.7** | 6.7 (2.2) | -0.6** | 6.6 (2.3) | -0.7** |
| Month 12 | 7.0 (2.3) | -0.6* | 6.3 (2.6) | -1.1** | 6.3 (2.6) | -1.0** | 6.3 (2.5) | -1.0** |

LS mean change from baseline, **p*<.05, ***p*<.001
